# New insights into public perceptions of cancer

**DOI:** 10.3332/ecancer.2013.349

**Published:** 2013-09-10

**Authors:** Corina W Ramers-Verhoeven, Gary L Geipel, Moira Howie

**Affiliations:** 1 Global Communications, Global Oncology Corporate Affairs, Eli Lilly and Company, 3991 RA, Houten, The Netherlands; 2 Global Oncology Corporate Affairs, Eli Lilly and Company, Indianapolis, Indiana 46285, USA; 3 Global Advocacy & Professional Relations, Oncology Eli Lilly and Company, Windlesham, Surrey, GU20 6PH, UK

**Keywords:** cancer, public perception, research, PACE, patient access, progress, public opinion, cancer perceptions, cancer treatment, cancer care

## Abstract

A survey was conducted to identify perceptions of cancer and its treatment among the general public, patients, and care givers in six countries. The purpose of the research—the PACE Cancer Perceptions Index (2012)—was to share public perspective on the progress of cancer care and treatment with stakeholders who make decisions about cancer innovation and access to treatments in order to allow patient-centric decisions. The results revealed that although understanding of cancer is increasing, a number of misconceptions persist. Although most respondents recognise that progress has been made in cancer treatment and a majority express satisfaction with this progress, they nevertheless want more investment and faster access to new cancer treatments. In particular, there was clear agreement that more collaboration was needed across countries and between the various stakeholders within countries. In addition, a clear majority of respondents does not think that their country invests sufficient funds in cancer research, and there is concern that progress may be jeopardised by the current difficult economic times.

## Introduction

Forty years after the US government declared a ‘War on Cancer’, and following decades of massive investment in cancer research and treatment throughout much of the world, it is an appropriate time to consider what can be done to improve the rate of progress against this disease. In 2012, Lilly Oncology formed a global network dedicated to encouraging policy changes that can accelerate research and development (R&D) in the cancer field and improve patient access to the most effective treatments, which is called PACE (for Patient Access to Cancer Care Excellence); this network includes stakeholders from diverse sectors involved in cancer R&D, care delivery, policy, and patient advocacy operating under a transparent action plan [1]. To inform the development of a reform agenda and to share insights with policy makers, PACE commissioned a rigorous opinion-research project focused on the general public as well as cancer patients and caregivers.

To better understand public attitudes about cancer and its treatments, PACE set out to establish a baseline of cancer knowledge and attitudes among the public in six countries. The results—the PACE Cancer Perceptions Index (2012) [2]—are presented in this short communication. The authors believe that progress on a number of key goals in cancer policy can be improved by referring to rigorous public-opinion analysis. For example, the goal of encouraging greater patient participation in cancer clinical trials is widespread, but efforts to achieve this goal rarely begin with an understanding of public perspectives on clinical research. Similarly, while many stakeholders appear to agree that decisions over treatment access and reimbursement need to be made in a patient-centric fashion, there is no consensus on how this should occur, and patient involvement in assessment processes remains in its infancy. Surprisingly few health technology assessment agencies use and invest in scientifically robust methods to gather evidence about the social and psychological aspects of living with an illness or using a technology [3]. Keeping patients at the centre of decision-making requires not only their direct involvement but also their greater understanding among other stakeholders about public and patients’ attitudes, needs, and priorities.

It is hoped that the insights from the PACE Cancer Perceptions Index will play a role in formalising the voice of the public on these and other cancer-related issues while also providing insights to be explored in follow-on public-opinion research.

## Methods

The primary goal of the survey was to provide useful data that help inform and educate stakeholders about public perceptions and expectations of cancer care/innovation in order to make patient-centric decisions. In total, 4,341 individuals, including the general population (3,009), cancer patients (663), and caregivers (669) were polled from 28 August to 4 October 2012. The general public samples are projectable to the adult populations of the countries but patient and caregiver samples were convenience samples intended to provide comparisons of patterns across the group.

Survey participants were from six countries: the United States, France, Germany, Italy, Japan, and the United Kingdom. These countries were selected for their significant influence in shaping broader policy models in cancer care. The survey was carried out by GfK, one of the leading research companies with extensive experience of research of this nature.

The general population surveys were achieved using random digital dial computer-assisted telephone probability techniques in each country. The sample comprised approximately 70% landline call respondents and 30% cell phone respondents in each country in order to account for the fact that some households only have cell phones. Samples of 500 respondents were targeted in each country. All interviews were conducted from London, and all interviewers were native speakers of the language of the country being called. Aside from questions to screen and elicit demographic information, the survey consisted of 26 questions. For the most part, the same questions were asked of all sample groups. However, in certain cases, the question was adapted for the patient and caregiver sample groups, for example, in relation to experience of clinical trials.

With the exception of the United States, in each field country, approximately 100 cancer patients and 100 caregivers were interviewed using an online survey substantially similar to the one administered to the general public. In the United States, approximately 150 patients and 150 caregivers were interviewed. The sample source for these surveys was primarily uSamp; in Germany, Survey Sampling Inc. (SSI) sample was also used. In Japan, four sample providers were used: uSamp, AIP, Toluna, and SSI. An overview of the sampling scheme is shown in Table 1. The achieved sample sizes are listed in the table.

The (weighted) mean age of the general population was 46 years, and in the patient and caregiver populations, it was 48 and 38 years, respectively. In this respect, it should be noted that patient samples were considerably younger than overall cancer patient populations, and their views may not therefore be reflective of older patients. The gender ratio of male to females was 49:51 in the general population versus 45:55 in the patient groups, and 50:50 for caregivers.

The general population data in all countries were weighted in order to balance the sample to national demographic statistics including region. Weighting data, while beneficial in better reflecting the sample population, does increase the margin of sampling error and in turn reduces the effective base upon which statistical tests are performed. The effective bases and margins of sampling error for the weight results for each country are shown in Table 2 for a statistic at 50% at the 95% confidence limit based upon the entire sample for a country. The margin of the sampling error decreases as the statistic gets farther from 50% (e.g., a finding at 30% has a smaller margin of sampling error associated with it than a finding at 50%). In addition, the margin of sampling error is higher and varies for results based on sub-samples. Table 2 displays the results.

## Results

### The situation today

Initially, the respondents were asked about their perspective on the fight against cancer and their satisfaction with the progress that has been made over the past 20 years. Nearly six in ten (57%) public respondents claimed to be very or somewhat satisfied with the progress made with highest satisfaction levels being reported in the United Kingdom; significantly higher levels of satisfaction were also expressed by patients and caregivers, 78% and 63%, respectively, said they were very or somewhat satisfied with the progress (5% risk level).

Moreover, nearly half (48%) of the general population disagreed with the statement ‘regardless of treatment, a cancer diagnosis will ultimately result in death’. There were, however, significant differences between countries in relation to this, with public respondents in the United States being most optimistic (65% strongly disagree or disagree with the statement) and those in Japan the least (only 36% strongly disagree or disagree) (*p *≤ 0.05). Nevertheless, when asked about their greatest concerns if they were personally to receive a diagnosis of cancer, 67% of public respondents said the impact on family and friends, 66% said death as a result of cancer, and 65% said paying for treatment.

In terms of the cancer treatment available to them, only 31% of general public responders were very or extremely confident in the cancer care provided by their respective health care systems although patients were much more likely than the general public—54% versus 31%—to be at least very confident that they received the best treatment (*p *≤ 0.05). Among patients across countries, information about the financial impact of cancer tops the list of unmet needs (55%), although navigating treatment options and emotional impact were not far behind.

### Oncology medicine development

The survey also revealed some misconceptions about cancer and its treatment. When asked whether cancer was one disease or many, 43% of public respondents said it was ‘a single disease that could appear in different parts of the body’ compared with 51% who agreed with the statement that ‘cancer was a different disease that can appear in different parts of the body’. However, most people did recognise that the same cancer medication could produce very different results in patients with similar diagnoses.

Other misconceptions related to the development of cancer medicines themselves in relation to both time taken and costs involved. The majority of people surveyed believe that cancer medicines go from discovery to prescription in ten years or less. In reality, the time taken to develop a new cancer medicine is between 10 and 15 years [4–6]. And interestingly, in spite of the fact that a clear majority underestimates the amount of time required to develop a medicine, most—71% of the general population—thought it took too long for new treatments to become available.

The public also significantly underestimates the cost of developing cancer treatment. Using roughly comparable currency categories, two in three public respondents or more—73% in the United States—across all the countries surveyed believe cancer medicine development costs fall within the lowest three categorical choices—100 million euros or less, 100 million pounds sterling, 100 million dollars, or 10 billion Japanese yen. Only a minority were aware that the costs reached one billion US dollars or more [7].

In terms of development of medicine, just over half of the public respondents appeared to understand the nature of innovation, with 56% agreeing that progress arose by ‘smaller but important advances that improved how long patients survive a little bit at a time,’ although 37% thought that progress arises as a result of ‘major breakthroughs that dramatically improve how long patients survive’.

### The future of cancer research

As well as assessing baseline understanding of cancer, medicines, and treatment, public respondents were asked about the future of cancer research. In every country—with the exception of France—a majority or near majority of respondents thinks its country invests too little in fighting the disease. In fact, half of the public and roughly two-thirds of patients and caregivers feel that too little money has been spent to fight cancer, with highest dissatisfaction among the public in Italy, where 58% say too little has been invested, and lowest in France, where 43% said too little had been invested.

There was a very high agreement on the need for greater collaboration across borders and among stakeholders, with 86% of the general population agreeing or strongly agreeing that cancer research and development efforts should be coordinated across national borders and 83% agreeing or strongly agreeing that greater collaboration among government, academia, and pharmaceutical companies is required to accelerate progress in cancer research.

Large majorities across all respondent groups saw academic researchers as pre-eminent in cancer medicine development, although more than half rank the pharmaceutical industry as a top player (other options included non-profit organisations and government agencies). However, although perception of the importance of the pharmaceutical industry was high, six in ten in all three groups thought those pharmaceutical companies were in general more motivated to treat cancer rather than cure cancer.

Furthermore, 62% of public respondents—reaching 70% in the United Kingdom—expressed concern that progress will be slowed because of the poor economy (*p* ≤ 0.05).

### Clinical trials

As one of the biggest challenges facing the development of new medicines is recruitment to clinical trials, the survey included some questions about attitudes to such trials. The results reveal that the public does perceive clinical trials as an opportunity to advance medical research and receive better treatments currently not available. For example, nearly three-quarters of public respondents said they would be willing to participate in clinical trials if they were a patient themselves, and it would either improve hope of receiving a life extending treatment or improve the likelihood of helping future patients (74% and 72%, respectively). However, from the patient group, few have participated in a clinical trial; inconvenience, concern about safety risks, and additional costs (e.g., changing physicians) work against participation.

### Personalised medicine

Survey participants were also asked about their attitudes to personalised medicine. As an introduction, it was explained that personalised medicine was an evolving approach to medicine that is being used in the treatment of cancer and other diseases. It went on to explain that it involved collecting biological information from the patient to help predict which treatment was likely to work, not work, or to cause side effects for that patient. Respondents were then asked whether they had heard about personalised medicine already; 34% of the general population said they had, and 64% said they had not. Awareness was highest in the United States (48%) and patients were more likely to have heard about personalised medicine but even so awareness levels remained quite low at 41% for all countries (*p* ≤ 0.05).

Once familiar with the concept, a large majority of respondents agreed that doctors should discuss personalised medicine with all cancer patients and said they would want to be tested for personalised medicine even if it was possible that they would learn that personalised treatment would not work for them.

One of the concerns about personalised medicine is around privacy relating to the sharing of medical records. Asked about health information technology, large majorities—more than eight out of ten respondents in all three groups said they would be happy to share their own medical records to help themselves and other patients; still, sizable proportions—44% of the general population—report concerns about potential misuse of data, with Italian public respondents expressing the most concern (53%) and Japanese public respondents expressing the least concern (35%) (*p* ≤ 0.05). The percentage in the United States is 48%.

### Cost of treatment

Finally, in light of ongoing debates about the costs of oncology treatment, the survey endeavoured to gauge public perception regarding valuation of one quality year of life. There was no consensus on how much money should be spent on treatment in exchange for an extra year of life. A sizable minority in European Union nations (40%) places a high value on this extra year (up to €200,000 or more). In the United States, 24% believe an extra year of life is worth as much as US$200,000 plus.

However, there is consensus on who should pay for life-prolonging treatments—72% say public payers/insurers. Furthermore, strong majorities of respondents want patients and families (78%), along with physicians (41%), to decide on these treatment options.

## Discussion

The findings from the PACE Cancer Perceptions Index provide interesting information about the public perception of cancer and serves as a useful starting point to better understand the public perspective. As a developer of cancer therapies, Lilly believes that bringing the perspectives of the public and patients to stakeholders, including policy makers and health care managers, into cancer care discussions will add a critical new dimension to ongoing discussions about access to treatments and the future of cancer care.

As with any research, the limitations of the methods must of course be taken into account. There are several caveats that limit interpretation of the data including the significance of language, the known inconsistencies between stated intentions and behaviours and the preferred reliance on behaviour as a more credible indicator of preferences, the lack of consensus about cost computations across countries, and potential biases as a result of leading questions.

Nevertheless, these findings do provide useful information on the patient perspective, and the voice of the patient can and should influence the dialog between groups with different agendas (e.g., insurers/payers and politicians).

The results indicate that while the public is aware of the magnitude of cancer innovation that has taken place, there is clear frustration that progress is not fast enough and concern that future progress may be slowed as a result of the challenging economic environment. Respondents see cancer as a clear health priority and want greater investment in tackling the disease and faster availability of medicines.

In particular, the findings suggest more should be done to facilitate patient participation in clinical trials. This was one of the areas in particular where findings are hard to reconcile. For example, while the survey showed that, in all countries, the majority of respondents are willing to participate in clinical trials, the fact remains that only 2%–3% actually enrols [8]. If low enrolment does not stem from patients’ unwillingness to participate, better understanding is needed regarding why patients choose not to participate (e.g., high costs/lack of insurance, concerns about substandard treatment or safety issues, inconvenience due to geographic distance, risks to employment, restrictive insurance inclusion/exclusion criteria—for example, displacement related to geography and employment can threaten insurability). Follow-on surveys can address these gaps in knowledge to help confront the challenges of clinical trial participation.

More insight is also needed to understand the value the public is prepared to place on treatment. In order to understand fair price and reimbursement, the factors that contribute to the calculation need to be clear. The survey showed that people continue to underestimate the costs of medicine development. Understanding patients’ attitude to value will require better understanding on their part of the true costs and efforts involved in developing medicines through education as well as additional future efforts to develop a clearer understanding of patients’ views.

Regrettably, the survey indicates the public’s continued mistrust of the pharmaceutical industry, and more work is needed on the part of industry to work in a more transparent way and provide greater information to address trust concerns.

In short, knowing patients and keeping their interests at the centre of all efforts is critical in these economically challenging times. This survey provides some useful information that can help stakeholders understand the public perspective and identifies areas where follow-on surveys can continue to discover and communicate public perceptions about topics related to cancer in general, cancer medicine/treatment development and innovation, research funding and sources, roles of different players in cancer medicine development, personalised medicine, health information technology, importance of clinical trials, and willingness to pay for innovative treatment options.

## Figures and Tables

**Table 1.** Lilly PACE survey populations and sample size summary.

Country	General public	Patients	Caregivers
Description	Nationally representative RDD sample 18+ (70% LL; 30% cell sample)	Have been doctor diagnosed with cancer within the past five years; must have received treatment (e.g., surgical, radiation, or chemotherapy)	Have been or are currently a caregiver for a family member or close friend who has been diagnosed with cancer in the past five years; person must have received treatment (e.g., surgical, radiation, or chemotherapy)
	**Sample size/country**	**Sample size/country**	**Sample size/country**
Germany	*n* = 500	*n *= 102	*n *= 102
France	*n* = 500	*n *= 100	*n *= 102
Italy	*n *= 503	*n *= 102	*n *= 103
United Kingdom	*n *= 501	*n *= 101	*n *= 104
Japan	*n *= 500	*n *= 105	*n *= 102
United States	*n *= 505	*n *= 153	*n *= 156
Total	*n *= 3009	*n *= 663	*n *= 669

**Table 2.** Lilly PACE general population sample sizes, effective bases, and margins of error.

General population RDD country	Sample size	Effective sample size	Margin of error at 50% statistic, 95% confidence interval
Germany	*n *= 500	431	±4.7%
France	*n *= 500	450	±4.6%
Italy	*n *= 503	450	±4.6%
United Kingdom	*n *= 501	379	±5.0%
Japan	*n *= 500	446	±4.6%
United States	*n *= 505	379	±5.0%
Overall	*n *= 3009	2,536	±1.9%
